# Mutations at the palmitoylation site of non-structural protein nsP1 of Semliki Forest virus attenuate virus replication and cause accumulation of compensatory mutations

**DOI:** 10.1099/vir.0.82865-0

**Published:** 2007-07

**Authors:** Eva Žusinaite, Kairit Tints, Kaja Kiiver, Pirjo Spuul, Liis Karo-Astover, Andres Merits, Inga Sarand

**Affiliations:** 1 Institute of Molecular and Cell Biology, University of Tartu, Nooruse 1, 50411 Tartu, Estonia; 2 Estonian Biocentre, Riia Street 23, 51010 Tartu, Estonia; 3 Institute of Biotechnology, University of Helsinki, 00014 Helsinki, Finland

## Abstract

The replicase of Semliki Forest virus (SFV) consists of four non-structural proteins, designated nsP1–4, and is bound to cellular membranes via an amphipathic peptide and palmitoylated cysteine residues of nsP1. It was found that mutations preventing nsP1 palmitoylation also attenuated virus replication. The replacement of these cysteines by alanines, or their deletion, abolished virus viability, possibly due to disruption of interactions between nsP1 and nsP4, which is the catalytic subunit of the replicase. However, during a single infection cycle, the ability of the virus to replicate was restored due to accumulation of second-site mutations in nsP1. These mutations led to the restoration of nsP1–nsP4 interaction, but did not restore the palmitoylation of nsP1. The proteins with palmitoylation-site mutations, as well as those harbouring compensatory mutations in addition to palmitoylation-site mutations, were enzymically active and localized, at least in part, on the plasma membrane of transfected cells. Interestingly, deletion of 7 aa including the palmitoylation site of nsP1 had a relatively mild effect on virus viability and no significant impact on nsP1–nsP4 interaction. Similarly, the change of cysteine to alanine at the palmitoylation site of nsP1 of Sindbis virus had only a mild effect on virus replication. Taken together, these findings indicate that nsP1 palmitoylation as such is not the factor determining the ability to bind to cellular membranes and form a functional replicase complex. Instead, these abilities may be linked to the three-dimensional structure of nsP1 and the capability of nsP1 to interact with other components of the viral replicase complex.

## INTRODUCTION

The alphaviruses (family *Togaviridae*) are enveloped, positive-strand RNA viruses that infect both vertebrate hosts and insect vectors (Strauss & Strauss, 1994). Due to their broad host range, high levels of protein expression and small genomes, alphaviruses have been extensively exploited as models. Semliki Forest virus (SFV) is one of the best-studied alphaviruses. The SFV genome is approximately 11.5 kb long and has a 5′ cap structure and 3′ poly(A) sequence. It encodes four non-structural proteins (nsP1–4), involved in viral RNA synthesis, and five structural proteins. After virus entry into the cell, the genomic RNA is translated into a large, non-structural polyprotein, which is processed to form an early and subsequently a late replicase complex (Strauss & Strauss, 1994). The early replicase complex mediates synthesis of the negative-strand RNA, which, in turn, is used by the late replicase complex as template for synthesis of new genomes and subgenomic RNA for structural proteins. Structural genes are not required for replication and thus can be replaced with a polylinker and/or with foreign sequences in order to obtain SFV-based replicon vectors (Liljestrom & Garoff, 1991).

The nsP1 protein is required for alphavirus RNA replication. It has been shown that nsP1 is required for synthesis of negative-strand RNAs (Wang *et al.*, 1991) and that nsP1 catalyses the capping reaction of viral RNAs (Ahola & Kaariainen, 1995; Wang *et al.*, 1996). It is also the only non-structural protein to interact with membranes (Ahola *et al.*, 1999; Salonen *et al.*, 2003). The membrane association of nsP1 is mediated through direct interaction of the amphipathic binding peptide (BP) with anionic phospholipids (Ahola *et al.*, 1999) and is increased by post-translational palmitoylation of between one and three cysteine residues at positions 418–420 (Laakkonen *et al.*, 1996). It has recently been demonstrated that nsP1 can only become palmitoylated after associating with membranes via the BP (Spuul *et al.*, 2007). The replacement of cysteines at the palmitoylation site with alanines abolishes palmitoylation of nsP1 completely, whereas the essential enzymic activities of the protein, as well as its ability to bind to cellular membranes, are not destroyed (Laakkonen *et al.*, 1996). It has also been reported that the cysteine-to-alanine mutations did not have a significant effect on SFV viability or growth in cell culture, although SFV_1PA−_ virus was avirulent in mice (Ahola *et al.*, 2000).

Here, we demonstrate that substitutions or deletions at the nsP1 palmitoylation site and its surrounding sequences seriously affect the viability of SFV. The subsequent replication of the mutant viruses to high titres was found to be due to the accumulation of second-site compensatory mutations, which restored the interaction between nsP1 and nsP4 that was abolished by the primary mutations at the palmitoylation site.

## METHODS

### Cells, virus strains and media.

BHK-21 cells were grown in Glasgow's minimal essential medium (GMEM) containing 5 % fetal calf serum (FCS), 10 % tryptose phosphate broth, 100 U penicillin ml^−1^ and 0.1 mg streptomycin ml^−1^. Cos-7 cells were grown in Iscove's modified Dulbecco's medium containing 10 % FCS, 100 U penicillin ml^−1^ and 0.1 mg streptomycin ml^−1^. HeLa cells were grown in Dulbecco's modified Eagle's medium containing 10 % FCS, 2 mM glutamine, 100 U penicillin ml^−1^ and 0.1 mg streptomycin ml^−1^. The SFV4 strain was derived from the infectious cDNA (icDNA) clone pSP6-SFV4 (Liljestrom & Garoff, 1991), and wild-type (wt) Sindbis virus (SIN) from the clone pTOTO1101 (Rice *et al.*, 1987). A derivative of SIN with replacement of cysteine at the palmitoylation site by alanine was derived from plasmid pSIN_1PA−_ (Ahola *et al.*, 2000). Modified recombinant vaccinia virus Ankara expressing T7 RNA polymerase (MVA-T7) was kindly provided by Bernard Moss (NIH, Bethesda, MD, USA).

### Construction of SFV mutants.

Replacement of the three cysteine residues at positions 418–420 with alanines (hereafter designated mut3A) and deletion of the three cysteine residues at positions 418–420, designated Δ3, or of the RSLTCCC sequence at positions 414–420, designated Δ7, were performed by PCR-based mutagenesis. PCR was performed on the pTSF1 template (Peranen *et al.*, 1993) and the DNA fragments with introduced mutations were cloned into a derivative of pSFV1 (Liljestrom & Garoff, 1991), pSFV1/d1EGFP, by exchange of the *Pfl*23II–*Sac*I fragment to obtain pSFV1mut3A/d1EGFP, pSFV1Δ3/d1EGFP and pSFV1Δ7/d1EGFP. Plasmids containing full-length icDNA (pSFV4mut3A, pSFV4Δ3 and pSFV4Δ7) were created by subsequent replacement of *Xba*I–*Spe*I fragments in the corresponding replicon plasmids by structural genes from pHelper1 (Liljestrom & Garoff, 1991). The replicons and genomes with identified compensatory mutations were obtained by similar cloning procedures.

### Construction of plasmids for transient expression.

The regions encoding nsP2 and nsP3 were excised from pTSF2 and pTSF3, respectively (Peranen, 1991), with appropriate restriction endonucleases. The coding sequence of SFV4 nsP4 was PCR amplified using pSP6-SFV4 as the template and primers containing appropriate restriction endonuclease sites. Fragments were cloned into pCG3F12 in frame with a bovine papillomavirus type 1 E2 protein-derived 3F12 epitope tag (Kaldalu *et al.*, 2000). The coding sequence of nsP1 was cloned into the same expression vector without the 3F12 tag. The resulting constructs were designated pCGnsP1, pCG3F12nsP2, pCG3F12nsP3 and pCG3F12nsP4. The mut3A, Δ3 and Δ7 mutations were introduced into pCGnsP1 by subcloning and the resulting plasmids were designated pCGmut3A, pCGΔ3 and pCGΔ7. Compensatory mutations, together with mut3A and Δ3 mutations, were subcloned into the pCGmut3A and pCGΔ3 vectors by the use of the unique restriction sites within the nsP1 sequence and the resulting clones were designated pCGmut3A/P181Q, pCGmut3A/L234F, pCGmut3A/Q357L, pCGΔ3/M124V+A197D and pCGΔ3/ΔG224+T352S.

For expression of nsP1 mutant variants in HeLa cells, the pTSF1 vector (Peranen *et al.*, 1993) was used. The mutations mut3A, Δ3 and Δ7, alone or together with corresponding compensatory mutations, were subcloned into pTSF1 from corresponding pCG vectors with *Eco*RV and *Mlu*I endonucleases. The resulting constructs were designated pTSFmut3A, pTSFΔ3, pTSFΔ7, pTSFmut3A/P181Q, pTSFmut3A/L234F, pTSFmut3A/Q357L, pTSFΔ3/M124V+A197D and pTSFΔ3/ΔG224+T352S.

### Construction of plasmids for bacterial expression.

The pBAT1 expression vector was used for expression of derivatives of nsP1 in *Escherichia coli* (Ahola *et al.*, 1997). The nsP1 sequences carrying mut3A, Δ3 and Δ7, alone or together with compensatory mutations, were transferred to pBAT1 from the corresponding pTSF1 vectors by *Nco*I–*Hin*dIII fragment exchange. The resulting clones were designated pBATmut3A, pBATΔ3, pBATΔ7, pBATmut3A/P181Q, pBATmut3A/L234F, pBATmut3A/Q357L, pBATΔ3/M124V+A197D and pBATΔ3/ΔG224+T352S.

### RNA transcription and transfection.

SFV-based replicons and icDNA plasmids were linearized by *Spe*I digestion; pTOTO1101 based plasmids were linearized by *Xho*I digestion. RNAs were synthesized by SP6 RNA polymerase and used for cell transfection by electroporation as described previously (Karlsson & Liljestrom, 2003). Primary virus stocks were collected from transfected cell cultures after incubation at 37 °C for 24 h.

### Virological methods.

Confluent monolayers of BHK-21 cell culture were washed with PBS, infected at an m.o.i. of 10 for 1 h at 37 °C and overlaid with complete medium. BHK-21 cells (8×10^6^ cells) were transfected with 50 μg *in vitro*-synthesized RNA or infected at an m.o.i. of 10 as described above. At selected time points, 150 μl medium was sampled and virus titres in the harvested samples were determined by plaque assay.

For the infectious centre assay, 1 μg *in vitro*-synthesized RNA was used for electroporation of 8×10^6^ BHK-21 cells. When needed, the amount of RNA transcripts used was increased to 10 μg. Tenfold dilutions of electroporated cells were seeded in six-well tissue-culture plates containing 1.5×10^6^ BHK-21 cells per well. After 2 h incubation at 37 °C, cells were overlaid with 2 ml carboxymethylcellulose (CMC) containing GMEM (2 % CMC : GMEM ratio 2 : 3, final concentration FCS 1.2 %). Plaques were stained with crystal violet after 2 days incubation at 37 °C.

For isolation of pseudorevertant viruses, 100 mm dishes of confluent BHK-21 cells were infected with primary stocks of the mutant viruses to obtain 20–50 plaques per plate. After 1 h incubation at 37 °C, cells were overlaid with 5 ml 0.9 % (w/v) agarose solution in GMEM and incubated for a further 2 days at 37 °C. To visualize plaques, the plates were overlaid with 3 ml 0.9 % (w/v) agarose/water solution containing 1 % (w/v) neutral red, and incubated overnight at 37 °C. Visible plaques were sampled by sterile tips and virions were eluted into GMEM. Stocks of viral clones were further amplified in BHK-21 cells.

### Mapping of compensatory mutations.

Viral RNA was purified by using the NucleoSpin RNA II system (Macherey-Nagel) and used for reverse transcription by RevertAid M-MuLV reverse transcriptase (Fermentas). The non-structural region of the SFV genome was PCR-amplified using a set of 13 pairs of primers and sequenced. Amplification and sequencing were repeated for fragments containing identified mutations.

### Co-immunoprecipitation.

Plasmids containing native or mutant sequences of SFV nsP1 were electroporated pairwise with plasmids pCG3F12nsP2, pCG3F12nsP3, or pCG3F12nsP4 (3 μg of each plasmid) into Cos-7 cells. Forty-eight hours post-transfection, the transfected cells were lysed in RIPA lysis buffer [50 mM Tris (pH 7.5), 400 mM NaCl, 1 mM EDTA, 1 % Igepal CA-630 (Sigma), 1 % sodium deoxycholate, 0.1 % SDS, 1 mM PMSF and EDTA-free protease inhibitor cocktail (Roche)] for 20 min on ice, incubated at −80 °C for 30 min and thawed on ice. The non-lysed fraction was removed by centrifugation at 15 000 ***g*** for 20 min. Protein concentration in cleared cell lysates was measured by Bradford assay. The amount of lysate corresponding to 1 mg total protein was incubated with nsP1-specific rabbit polyclonal antibody overnight at 4 °C. The immune complexes were precipitated with protein A–Sepharose CL-4B (Amersham Biosciences), pre-equilibrated in TNE buffer [50 mM Tris (pH 7.5), 400 mM NaCl, 1 mM EDTA]. Sepharose beads were washed four times with TNE buffer and precipitated proteins were denatured by boiling for 5 min in 50 μl Laemmli buffer. The amount of sample corresponding to 250 μg total protein was subjected to SDS-PAGE (12 % gel). Proteins were transferred to a nitrocellulose membrane, probed with the mouse 3F12 tag-specific monoclonal antibody (Kaldalu *et al.*, 2000) and visualized by using the ECL system (Amersham Biosciences).

### Immunofluorescence microscopy and palmitoylation assay.

Indirect immunofluorescence microscopy of transfected cells was carried out essentially as described by Salonen *et al.* (2003). Palmitoylation assay of the transiently expressed proteins was carried out as described by Spuul *et al.* (2007).

### Expression of recombinant proteins in *E. coli* and guanylyltransferase (GT) assay.

*E. coli* strain BL21 (DE3) was transformed with the expression plasmid pBAT1 containing wt or mutant nsP1 sequences. Conditions for cell growth and recombinant protein expression have been described elsewhere (Laakkonen *et al.*, 1994). Cells from 50 ml bacterial culture were collected at 4000 ***g*** for 10 min, resuspended in 1 ml lysis buffer [50 mM Tris/HCl (pH 8.0), 50 mM NaCl, 20 % glycerol, 0.1 % Tween 20, 1 mM DTT, 1 mM PMSF] and sonicated. The lysate was cleared by centrifugation at 15 000 ***g*** for 20 min at 4 °C, and the resulting supernatant (S15 fraction) was used for GT reaction or Western blotting. The covalent guanylyl complex formation reaction was carried out for 20 min at 30 °C in a mixture (total volume 30 μl) that contained 5 mCi (185 MBq) [*α*-^32^P]GTP (400 Ci mmol^−1^) in 50 mM Tris/HCl (pH 7.5), 10 mM KCl, 2 mM MgCl_2_, 5 mM DTT, 100 mM AdoMet (Sigma) and 5 μl sample (Ahola & Kaariainen, 1995).

## RESULTS

### Mutations at the nsP1 palmitoylation site affect SFV viability

**Fig. 1. f1:**
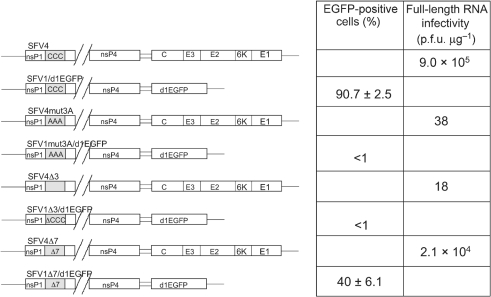
Schematic presentation and infectivity of SFV-based replicons and genomes with mutations at the palmitoylation site of nsP1. The infectivity of SFV-based replicons was measured as a percentage of EGFP-positive cells in 8×10^6^ BHK-21 cells transfected with 50 μg RNA. The mean±sd of three experiments are shown. Infectivity of full-length SFV genomes was measured by infectious centre assay; the experiment was repeated twice with similar results.

Three SFV-based replicons, containing mutations at the palmitoylation site and expressing d1EGFP as a marker, were constructed and analysed (Fig. 1). Transfection of BHK-21 cells revealed that <1 % of cells transfected with SFV1mut3A/d1EGFP or SFV1Δ3/d1EGFP RNAs were d1EGFP-positive, whereas transfection with SFV1Δ7/d1EGFP RNA resulted in approximately 40 % of cells expressing d1EGFP. Under the same conditions, the percentage of d1EGFP-positive cells was >90 % in cultures transfected with SFV1/d1EGFP. This severe effect of mutations at the palmitoylation site on the replication ability and marker gene expression of SFV-based replicons was unexpected, as similar changes in the context of the full-length SFV genome have previously been reported not to have a serious influence on SFV viability (Ahola *et al.*, 2000). To clarify the situation, all three mutations were transferred into pSP6-SFV4 and the infectivity of the corresponding RNA transcripts was analysed by infectious centre assay. It was found that the infectivity of SFV4mut3A and SFV4Δ3 RNAs was four orders of magnitude lower than that of SFV4 RNA, whereas the RNA infectivity of SFV4Δ7 was reduced by only about 40-fold (Fig. 1). Thus, both mut3A and Δ3 dramatically reduced infectivity of the corresponding transcripts, with Δ7 having a milder effect.

### Infectivity of the mutated SFV is restored during a single passage in cell culture

**Fig. 2. f2:**
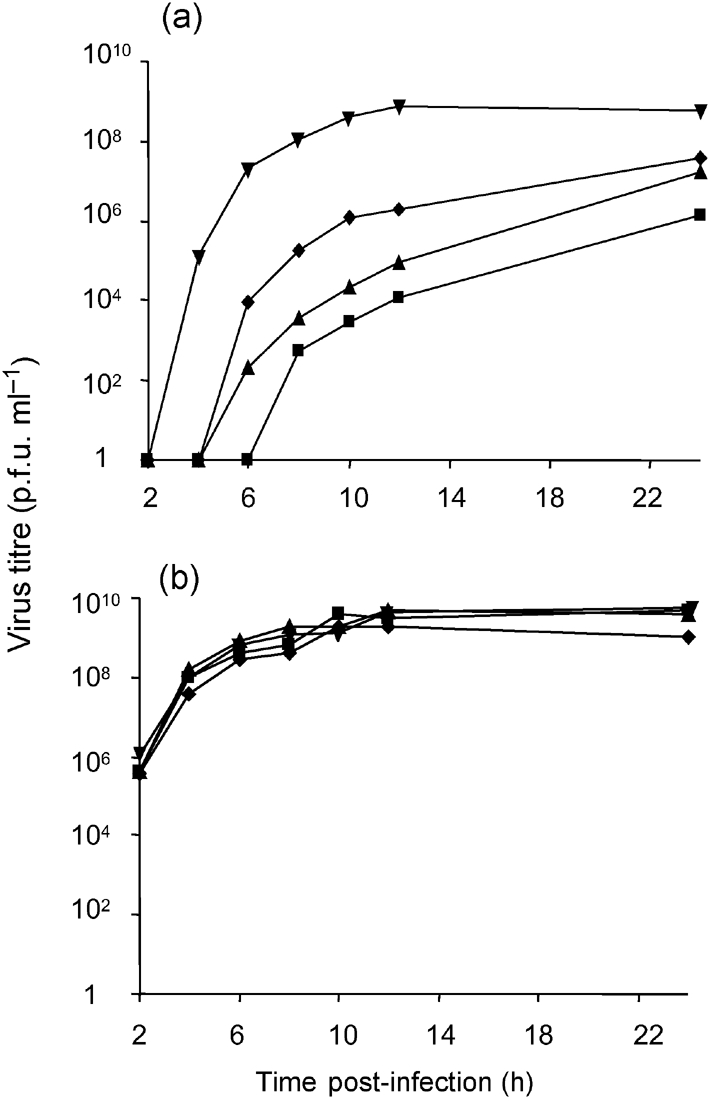
Growth curves of SFV4 (▾), SFV4mut3A (▴), SFV4Δ3 (▪) and SFV4Δ7(⧫) in 8×10^6^ BHK-21 cells upon (a) transfection with 50 μg *in vitro*-synthesized RNAs or (b) infection at an m.o.i. of 10. Data from one of two reproducible experiments are shown.

The most likely explanation for the seeming discrepancy between the effect of mut3A on virus replication described above and by Ahola *et al.* (2000) is the accumulation of changes with a positive impact on virus replication during virus propagation. To verify this hypothesis, we compared the release of viruses from transfected BHK-21 cells with that from BHK-21 cells infected with the corresponding viruses. It was found that the growth of the SFV4mut3A, SFV4Δ3 and SFV4Δ7 in transfected cells was delayed significantly compared with that of SFV4 (Fig. 2a[Fig f2]). The release of infectious virions was detected at 4 h post-transfection for SFV4; in contrast, the titres of the mutant viruses were below detection limits up to 6–8 h post-transfection. Consistent with previous findings, SFV4Δ7 showed the quickest growth among the mutants. The titres of SFV4 reached their maximum (10^9^ p.f.u. ml^−1^) 10 h post-transfection, whereas the titres of mutated viruses were always lower and continued to grow even at 24 h post-transfection (Fig. 2a[Fig f2]). When BHK-21 cell cultures were infected with the primary stocks of SFV4, SFV4mut3A, SFV4Δ3 or SFV4Δ7, no significant difference between the growth curves of these viruses was detected (Fig. 2b[Fig f2]). Thus, our results support the hypothesis that some changes took place during propagation of these mutant viruses.

### Mutation of the nsP1 palmitoylation site has a mild effect on SIN viability and replication

In order to study the effect in another alphavirus, the infectious centre assay was also performed with transcripts originating from pTOTO1101 and pSIN_1PA−,_ the mutant construct where the only cysteine residue at the nsP1 palmitoylation site is substituted with alanine (Ahola *et al.*, 2000). It was found that the mutation at the palmitoylation site reduced the infectivity of SIN transcripts by only about 30-fold. In growth-curve experiments, the release of infectious virions from cells transfected with transcripts from pTOTO1101 or pSIN_1PA−_ was, in both cases, detected at 4 h post-transfection, and only a small (<1 log) reduction in the titre of SIN_1PA−_, compared with that of SIN, was observed between 4 and 10 h post-transfection; at later time points, no statistically significant difference between titres of these viruses was observed (data not shown). These results are consistent with the previous report that the mutation at the palmitoylation site of nsP1 has only a minor effect on the viability and replication of SIN (Ahola *et al.*, 2000) and, accordingly, no compensatory changes are required to restore the replication of the mutant SIN genome.

### Identification of putative compensatory mutations in SFV

RT-PCR and sequencing analysis confirmed the presence of original mutations at the palmitoylation site of nsP1 for all three recombinant SFV stocks. To identify the possible second-site compensatory mutations, four isolates for each mutant were plaque-purified from the primary stocks of SFV4mut3A, SFV4Δ3 and SFV4Δ7, and their non-structural regions were analysed. This analysis revealed three single mutations (P181Q, L234F and Q357L) and two double mutations (M124V+A197D and ΔG224+T352S) in the SFV4mut3A and SFV4Δ3 clones, respectively (Table 1). All of these mutations, except ΔG224, were single-nucleotide replacements and localized either in the methyltransferase/guanylyltransferase (MT/GT) domain (M124V, P181Q, L234F, A197D and ΔG224) or in the C-terminal region (T352S and Q357L) of nsP1. In contrast to SFV4mut3A and SFV4Δ3, no mutations were found in the non-structural region of any plaque-purified isolate originating from SFV4Δ7.

### Identified mutations rescue the infectivity of SFV4mut3A and SFV4Δ3

The identified mutations were introduced into SFV4mut3A (P181Q, L234F and Q357L) or SFV4Δ3 (M124V+A197D and ΔG224+T352S) and their effects were analysed as for the original mutants. It was found that the introduction of these mutations increased the infectivity of *in vitro*-synthesized RNAs to a level comparable with that of SFV4 RNA (Table 1). Thus, the identified mutations did rescue the infectivity of SFV harbouring mut3A or Δ3 mutations.

Changes identified in SFV4Δ3 isolates were double mutations (M124V+A197D and ΔG224+T352S). When they were introduced separately into pSFV4Δ3, the subsequent analysis revealed that only one mutation from each pair (A197D and ΔG224, respectively) provided a compensatory effect, whereas the other was functionally neutral (Table 1).

**Fig. 3. f3:**
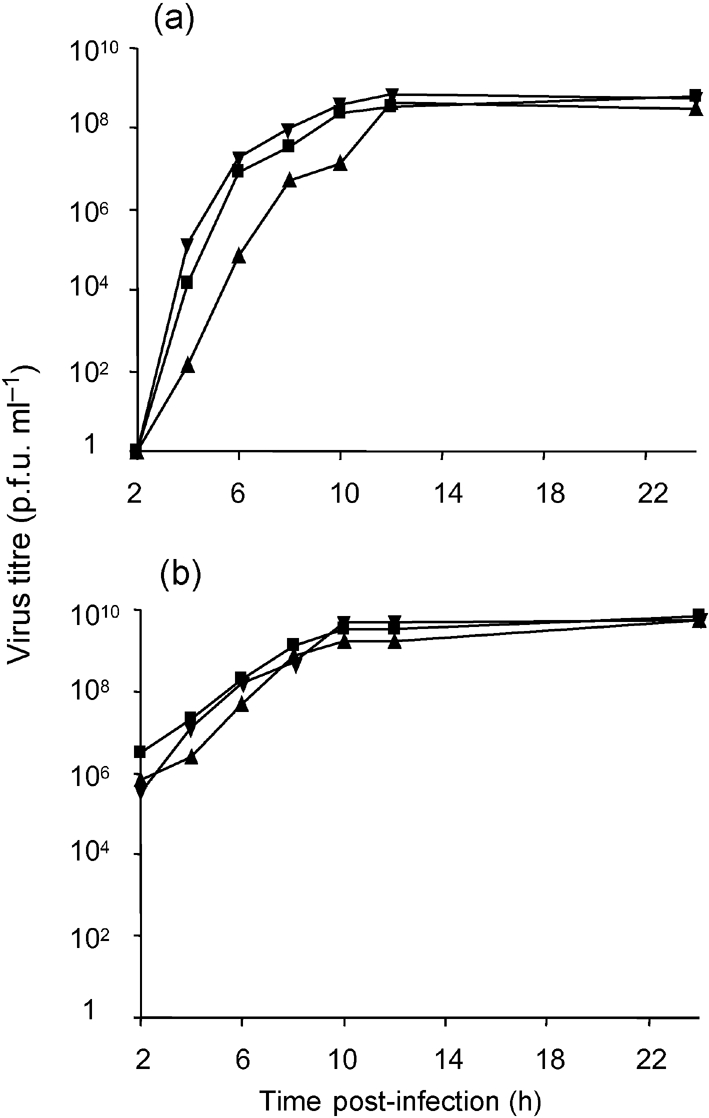
Growth curves of SFV4 (▾), SFV4mut3A-P181Q (▴) and SFV4Δ3-ΔG224+T352S (▪) in BHK-21 cells upon (a) transfection with 50 μg *in vitro*-synthesized RNAs or (b) infection with primary stock virus at an m.o.i. of 10. Data from one of two reproducible experiments are shown.

In order to demonstrate that compensatory mutations are also responsible for the differences between primary and secondary growth curves (Fig. 2), similar growth curves for two recombinants, SFV4mut3A-P181Q and SFV4Δ3-ΔG224+T352S, were constructed. It was found that, unlike SFV4mut3A and SFV4Δ3, the growth of SFV4mut3A-P181Q and SFV4Δ3-ΔG224+T352S in transfected BHK-21 cells had no delay in virion release, although their accumulation was slightly slower than that of SFV4 (Fig. 3a[Fig f3]). When BHK-21 cells were infected with the primary viral stocks of SFV4mut3A-P181Q and SFV4Δ3-ΔG224+T352S, no differences from SFV4 were detected (Fig. 3b[Fig f3]).

### Effects of compensatory mutations in SFV4

The compensatory mutations were introduced into SFV4 and their effects were analysed as described above. The only change detected in this experiment was a slight decrease in the infectivity of transcripts harbouring these mutations (Table 2). Thus, the identified compensatory mutations are almost-neutral mutations in the SFV4 background.

Next, the question of whether the compensatory mutations were effective only in their original background was examined. For that purpose, mutation L234F was introduced into genomes harbouring Δ3 or Δ7 deletions and analysed as described above. The results indicated that the L234F was able to compensate defects caused by the Δ3 mutation (Table 2). In contrast, the L234F mutation decreased the infectivity of full-length RNA of SFV4Δ7 by approximately 20-fold (Table 2). Based on these results, it can be suggested that the consequences of mut3A and Δ3 have a rather similar effect on nsP1 and can therefore be compensated by the same mutation(s). In contrast, the Δ7 mutation has a different effect.

### Enzymic activities and palmitoylation of mutant forms of nsP1

**Fig. 4. f4:**
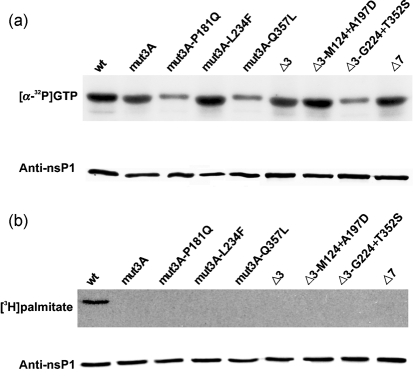
Guanylyltransferase activity and palmitoylation of wt and mutant nsP1 proteins. (a) Guanylyltransferase activity of recombinant nsP1s. Western blots of the S15 fractions of bacterial extracts were probed with anti-nsP1 antiserum. The same extracts were incubated with [*α*-^32^P]GTP in the presence of 100 mM AdoMet, and analysed by SDS-PAGE and autoradiography to reveal the covalent guanylate complexes. (b) Palmitoylation of transiently expressed nsP1s. HeLa cells were infected with MVA-T7, transfected with the respective pTSF1 constructs and labelled with [^3^H]palmitate. Western blots of HeLa extracts were probed with anti-nsP1 antiserum. Equal amounts of the same extracts were immunoprecipitated with anti-nsP1, separated by SDS-PAGE and autoradiographed to reveal the [^3^H]palmitate labelling of nsP1 variants.

It has been reported that the CCC to AAA (418–420) mutation has a relatively minor effect on the enzymic activities of recombinant nsP1 (Laakkonen *et al.*, 1996). To find out whether that is the case for nsP1s harbouring mut3A, Δ3 and Δ7 mutations or compensatory mutations together with mut3A and Δ3 mutations, proteins were expressed in *E. coli* and their GT activities were assayed. As is evident from the data presented in Fig. 4(a), the GT activity of recombinant proteins with mut3A, Δ3 and Δ7 was very similar to that of wt nsP1 and all recombinant proteins with compensatory mutations had GT activity as well. As the GT activity of nsP1 is completely dependent on its MT activity (Ahola & Kaariainen, 1995), the data indicate that the analysed proteins should possess MT activity as well. Thus, it can be concluded that defects (if any) in enzymic activities of mutant nsP1s could not represent the main reason for the very low infectivity of mut3A and Δ3 constructs.

As the enzymic activities of nsP1 are strictly dependent on its association with membranes (Ahola *et al.*, 1999), the data presented above clearly indicate that all recombinant nsP1s must be membrane-bound. This finding is consistent with the model that the primary binding of nsP1 to the membranes is mediated by the BP and that only then is it strengthened by covalent palmitoylation (Spuul *et al.*, 2007). All three mutations analysed in this study were expected to abolish palmitoylation of nsP1. The reversion of original mutations was never observed in this study, indicating strongly that palmitoylation of nsP1 was not restored. This hypothesis was verified directly by expression of mutant nsP1s by use of the MVA-T7 system in HeLa cells and the labelling of recombinant proteins with [9,10(n)-^3^H]palmitic acid. This experiment revealed that only wt nsP1 was palmitoylated (Fig. 4b[Fig f4]), confirming that mut3A, Δ3 and Δ7 did indeed abolish palmitoylation and that the compensatory mutations did not restore that function.

### Subcellular localization of mutant forms of nsP1

**Fig. 5. f5:**
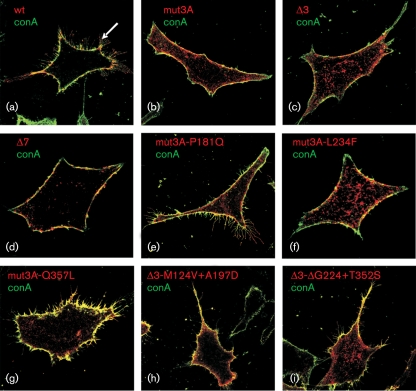
Subcellular localization and membrane binding of wild-type and mutant nsP1 proteins. HeLa cells infected with MVA-T7 were transfected with plasmid encoding the indicated nsP1 variants. The cells were fixed and stained with Alexa488-conjugated concanavalin A (conA; green) to visualize the plasma membrane, and then permeabilized for Alexa568–anti-nsP1 staining (red). Co-localization of conA and nsP1 is seen in yellow. The arrow in (a) indicates filopodium-like structures.

The analysis of the subcellular localization of mutant nsP1s in transfected HeLa cells by the MVA-T7 system revealed that, as expected, the wt nsP1 was localized almost exclusively at the plasma membrane and caused extensive induction of filopodium-like extensions (Fig. 5a[Fig f5]). In contrast, nsP1-mut3A and nsP1-Δ3 localized both on the plasma membrane and in the cytoplasm of transfected cells and caused no (or very little) formation of filopodium-like extensions (Fig. 5b, c[Fig f5]). Expression of nsP1-Δ7 also did not result in formation of filopodium-like extensions but, in contrast to nsP1-mut3 and nsP1-Δ3, the nsP1-Δ7 localized mostly at the plasma membrane of transfected cells (Fig. 5d[Fig f5]). Addition of the compensatory mutations to nsP1-mut3 and nsP1-Δ3 always resulted in more extensive plasma-membrane localization of the recombinant proteins (Fig. 5e–i[Fig f5]); this phenomenon is clearest in the cases of nsP1-mut3A-Q357L (Fig. 5g[Fig f5]) and nsP1-Δ3-M124V+A197D (Fig. 5h[Fig f5]). With the notable exception of nsP1-mut3A-L234F (Fig. 5f[Fig f5]), all of the recombinant proteins with compensatory mutations also induced the formation of filopodium-like extensions, although these structures were always less prominent than those induced by wt nsP1. Thus, the effects of compensatory mutations on the formation of filopodium-like extensions differ from each other. However, it should be noted that all viruses with compensatory mutations in their genome (but not SFV4-Δ7) caused the formation of filopodium-like extensions on the plasma membrane of infected BHK-21 cells, indicating that this phenomenon may depend on the mode of nsP1 expression and/or on the host cell type.

### Mutations at the palmitoylation site of nsP1 affect its interaction with nsP4

**Fig. 6. f6:**
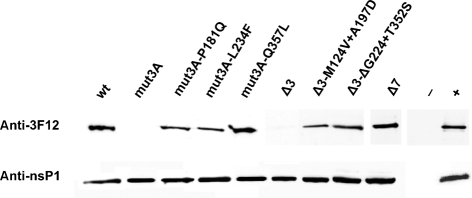
Interaction of wt and mutant forms of nsP1 with nsP4. Cos-7 cells were pairwise transfected with pCG plasmids expressing nsP4 and wt or mutated nsP1. Cells were collected 48 h post-transfection, lysed and proteins were precipitated with nsP1-specific antibody. The immunoprecipitated fraction was analysed by SDS-PAGE, and the presence or absence of co-immunoprecipitated nsP4 (with N-terminal 3F12 tag) was revealed by Western blotting by the use of 3F12 tag-specific mouse monoclonal antibody. Western blots of 10 μg total lysate of cotransfected Cos-7 cells were probed with anti-nsP1 antiserum. − and + represent 10 μg total protein of mock-transfected Cos-7 cells and Cos-7 cells transfected with pCG3F12-nsP4, respectively.

It has been reported that nsP1 interacts directly at least with nsP3 and nsP4, and that these interactions can be detected even if these proteins are expressed pairwise in a transient system (Salonen *et al.*, 2003). To determine the effects of mut3A, Δ3 and Δ7 on interactions of nsP1 with other non-structural proteins, nsP1, nsP1-mut3A, nsP1-Δ3 and nsP1-Δ7 were expressed pairwise with nsP2, nsP3 or nsP4 in Cos-7 cells and complexes were immunoprecipitated under native conditions with the anti-nsP1 antibody. The results of this experiment failed to reveal any difference between wt and mutated nsP1 regarding their interactions with nsP2 and nsP3, which were negative and positive, respectively (data not shown). However, in contrast to wt nsP1 and nsP1-Δ7, nsP1-mut3A and nsP1-Δ3 failed to interact with nsP4 (Fig. 6). Importantly, it was also demonstrated that the introduction of the compensatory mutations P181Q, L234F or Q357L into nsP1-mut3A and mutations M124V+A197D or ΔG224+T352S into nsP1-Δ3 restored their interaction with nsP4 (Fig. 6). These findings suggest the possibility that one of the reasons for the low infectivity of SFV4mut3A and SFV4Δ3 was the lack or significant debilitation of interaction between nsP1 and nsP4 and that the compensation occurred through its restoration.

## DISCUSSION

Cysteines at positions 418–420 are critical for the palmitoylation of SFV nsP1 (Laakkonen *et al.*, 1996). In this work, we have shown that the replacement of these cysteines by alanines or their deletion was detrimental to recombinant RNA infectivity, whilst the more extended deletion comprising residues 414–420 caused a less dramatic effect. It appeared that the accumulation of SFV4mut3A and SFV4Δ3 to high titres in the second-passage virus stock was due to the appearance of compensatory mutations either in the MT/GT domain or in the C-terminal part of nsP1. None of the compensatory mutations found in this study restored the palmitoylation of recombinant proteins, confirming that the palmitoylation of nsP1 is not strictly required for SFV replication (Ahola *et al.*, 2000). The discrepancy between the results described above and those reported previously is likely to be due to the heterogeneous nature of the mutant virus stock used in the earlier study.

The rapid accumulation of compensatory mutations requires very low initial infectivity of the original mutant RNA. In this case, the infection will start from a small fraction of transfected cells, followed by the release of virus and *de novo* infection of initially uninfected cells, thus providing conditions for the selection of mutations with a positive impact on virus growth. No compensatory mutation in the non-structural region of plaque-purified isolates of SFV4Δ7 was identified in this study; the sufficiently high infectivity of SFV4Δ7 prevented the takeover of the original genotype by pseudo-revertants during single-cycle infection. The change of the single cysteine to alanine at the palmitoylation site of SIN nsP1 had only a very mild effect on virus multiplication in transfected cells. The changes were even smaller than those observed for SFV4Δ7 and, therefore, as for SFV4Δ7, no compensatory changes are required for SIN_1PA−_. It is tempting to speculate that the mild effect in the case of SIN may be due to the smaller change in the protein (only one cysteine residue is mutated); however, the results obtained for SFV4Δ7 indicate that there is no direct correlation between the size of the mutated region and the corresponding phenotypic change.

It is evident from our data that the lack of nsP1 palmitoylation is not the main reason for the low infectivity of SFV4mut3A and SFV4Δ3. Similarly, this defect cannot be attributed to defects in GT/MT activities, as the corresponding recombinant proteins were enzymically active. In addition, although most of the identified compensatory mutations were located within the MT/GT domain of nsP1, none of them caused a significant increase in enzymic activity of the corresponding recombinant protein (Fig. 4a[Fig f4]).

Direct interactions of nsP1 with nsP3 and nsP4 have been shown previously in co-immunoprecipitation experiments (Salonen *et al.*, 2003). The importance of interactions between nsP1 and the N terminus of nsP4 for the recognition of the promoter for minus-strand RNA synthesis has been revealed in SIN replication (Shirako *et al.*, 2000). Another genetic study also supported nsP1–nsP4 interaction (Fata *et al.*, 2002). Here, we demonstrated that mutations mut3A and Δ3 destroyed or debilitated the interaction between nsP1 and nsP4, possibly resulting in defect(s) in the assembly of the replication complex. Interestingly, the Δ7 mutation did not preclude the interaction of nsP1 with nsP4. A possible explanation could be that the conformational changes in the nsP1 three-dimensional structure, caused on the one hand by mut3A or Δ3 mutations and on the other hand by Δ7 mutation, are different. This conclusion is supported by the finding that compensatory mutation L234F, identified from an SFV4mut3A isolate, could compensate Δ3, but was deleterious for Δ7.

The presence of enzymic activity and plasma-membrane localization indicate that the lack of palmitoylation does not prevent the membrane binding of the corresponding mutant nsP1s. However, some changes in subcellular localization of nsP1 were observed for both nsP1-mut3A and nsP1-Δ3: they are less abundant at the plasma membrane and partially localized in the cytoplasm of transfected cells. As the third mutant protein, nsP1-Δ7, as well as proteins with compensatory mutations, had more extensive plasma-membrane localization (Fig. 5), it can be speculated that the defect in membrane association and/or subcellular targeting may be another mechanism underlying the low infectivity of these recombinant viruses. Indeed, if mut3A and Δ3 were to result only in defects in the interaction between nsP1 and nsP4, it would be logical to assume that compensatory mutations could take place in either of the proteins. However, we were unable to find any mutations in the nsP4 region either in the plaque-purified viruses described above or in a larger number of isolates, purified and analysed specifically for this purpose (data not shown). The most likely explanation for this is that mut3A or Δ3 led to multiple defects in the localization and functions of nsP1 and only changes in the same protein can compensate for these defects.

Mutations preventing the palmitoylation of nsP1 are reported to prevent the formation of filopodium-like extensions in infected or transfected cells (Ahola *et al.*, 2000; Laakkonen *et al.*, 1998). The biological significance of filopodium formation is not known but, nevertheless, it represents a notable characteristic of SFV infection. Therefore, it is interesting to note that HeLa cells transiently expressing nsP1-mut3A-P181Q, nsP1-mut3A-Q357L, nsP1-Δ3-M124V+A197D and nsP1-Δ3-ΔG224+T352S did have filopodium-like extensions on the plasma membrane, indicating that nsP1 palmitoylation is not strictly required for the formation of these structures. In contrast to other compensatory mutations, the L234F change did not restore the formation of filopodium-like extensions; therefore, it is possible that these mutants can be used for studies of the functional significance of this poorly understood phenomenon. However, it should be mentioned that, in BHK-21 cells infected with the corresponding viruses, the morphological differences were much less prominent, indicating possible significance of the expression mode (infection versus transient expression), presence or absence of other non-structural proteins and/or host cell type for this phenomenon.

Taken together, our results support the idea that palmitoylation of nsP1 as such is not required for efficient replication, nor does it determine the interaction between nsP1 and nsP4 or the initial plasma-membrane localization of nsP1. Instead, it is more likely that the effects of mutations can be attributed to conformational changes caused by the deletions/substitutions at the palmitoylation site.

## Figures and Tables

**Table 1. t1:** Localization of compensatory mutations and their effect on the infectivity of SFV genomes with the mut3A or Δ3 mutations

**Original mutation in nsP1**	**Compensatory mutation found**	Infectivity of full-length RNA (p.f.u. μg^−1^)*
None	–	9×10^5^
CCC→AAA (418–420)	P181Q	6×10^5^
CCC→AAA (418–420)	L234F	1×10^5^
CCC→AAA (418–420)	Q357L	6×10^5^
ΔCCC (418–420)	M124V+A197D	3×10^5^
	M124V	<10
	A197D	1×10^5^
ΔCCC (418–420)	ΔG224+T352S	9×10^5^
	ΔG224	6×10^5^
	T352S	<10

*Infectivity of full-length SFV genomes was measured by infectious centre assay. The numbers shown represent data from one of two experiments with similar results.

**Table 2. t2:** Effects of compensatory mutations on the infectivity of SFV4 genome

**Alteration in the palmitoylation site of nsP1**	**Compensatory mutation introduced**	Full-length RNA infectivity (p.f.u. μg^−1^)*
None	None	9.0×10^5^
None	P181Q	7.8×10^5^
None	L234F	1.3×10^6^
None	Q357L	1.2×10^6^
None	M124V+A197D	4.4×10^5^
None	ΔG224+T352S	2.0×10^5^
ΔCCC (418–420)	L234F	5.7×10^5^
Δ RSLTCCC (414–420)	L234F	1.0×10^3^

*Infectivity of full-length SFV genomes was measured by infectious centre assay. The numbers shown represent data from one of two experiments with similar results.
